# A Novel Approach to Describe the Time–Temperature Conversion among Relaxation Curves of Viscoelastic Materials

**DOI:** 10.3390/ma13081809

**Published:** 2020-04-11

**Authors:** Adrián Álvarez-Vázquez, Alfonso Fernández-Canteli, Enrique Castillo Ron, Pelayo Fernández Fernández, Miguel Muñiz-Calvente, María Jesús Lamela Rey

**Affiliations:** 1 Department of Construction and Manufacturing Engineering, University of Oviedo, 33203 Gijón, Spain; afc@uniovi.es (A.F.-C.); fernandezpelayo@uniovi.es (F.P.); munizcmiguel@uniovi.es (M.M.-C.); mjesuslr@uniovi.es (M.J.L.); 2 Royal Academy of Engineering of Spain, don Pedro 10, 28005 Madrid, Spain; enrique.castillo@unican.es; 3 Royal Academy of de Ciencias Exactas, Físicas y Naturales, Valverde 24, 28005 Madrid, Spain

**Keywords:** viscoelastic behavior, relaxation curves, master curve, shift factors, time–temperature superposition principle

## Abstract

Time and temperature, besides pressure in a lesser extent, represent the most significant variables influencing the rheological behavior of viscoelastic materials. These magnitudes are each other related through the well-known Time–Temperature Superposition (TTS) principle, which allows the master curve referred to relaxation (or creep) behavior to be derived as a material characteristic. In this work, a novel conversion law to interrelate relaxation curves at different temperatures is proposed by assuming they to be represented by statistical cumulative distribution functions of the normal or Gumbel family. The first alternative responds to physical considerations while the latter implies the fulfillment of extreme value conditions. Both distributions are used to illustrate the suitability of the model when applied to reliable derivation of the master curve of Polyvinil–Butyral (PVB) from data of experimental programs. The new approach allows not only the TTS shift factors to be estimated by a unique step, but the whole family of viscoelastic master curves to be determined for the material at any temperature. This represents a significant advance in the characterization of viscoelastic materials and, consequently, in the application of the TTS principle to practical design of viscoelastic components.

## 1. Introduction

**Figure 1:**
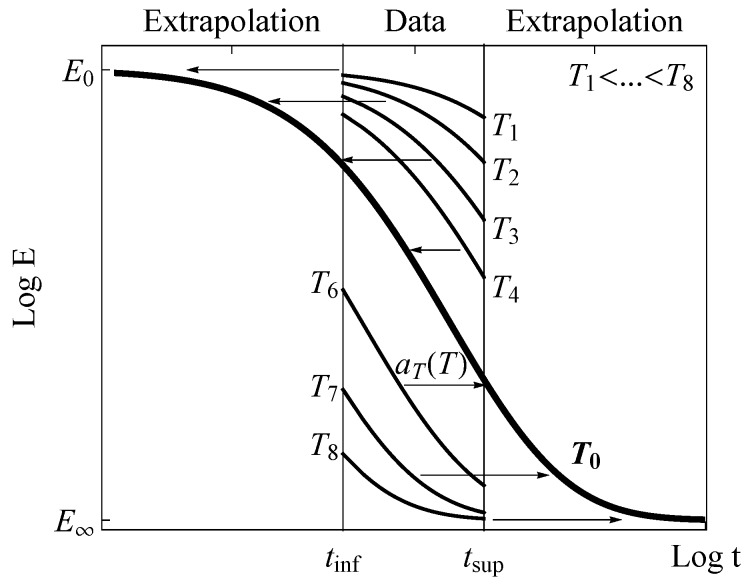
Scheme of the Time–Temperature Superposition (TTS) principle for the building master curve from short-time tests at different temperatures.

The characterization of viscoelastic materials represents a usual task in the current practical engineering to provide the basis of components design in a wide range of fields encompassing different materials such as polymers, biological materials and tissues, cementitious materials and woods. The mechanical behavior of these materials depends not only on time and frequency (see Findley et al. [1], Ferry [2], Tschoegl [3] and Lakes [4]), but is also influenced by multiple external effects, such as temperature, pressure and humidity. Among the different types of viscoelastic characterization (see Lakes [4]), the relaxation test allows the relaxation master curves of the material to be derived and becomes one of the favorite candidates due to its easy performance. Since the behavior of viscoelastic materials is time-dependent, their experimental tests would imply, in principle, long spaces of time to achieve the complete mechanical characterization over all their possible working time range, i.e., along several time decades (see Figure 1). This inconvenience can be overcome by resorting to the Time–Temperature Superposition (TTS) principle to characterize such thermo-rheologically simple viscoelastic materials (see Ferry [2] and Tschoegl [3]). Based on this principle, it is possible to compose the so-called master curve referred to a certain reference temperature from short-duration test records performed at different temperatures between two pre-established limiting lower and upper times $t_{1}$ and $t_{2}$, respectively, which determine the time interval during which test data are registered (see Figure 1). For example, the relaxation modulus of a material observed over a short-time test performed at a high temperature, say $T_{6}$ in Figure 1, would coincide with the corresponding relaxation modulus obtained over a long-time test at a reference temperature $T_{0}$. In this example, the short-time curve at $T_{6}$ must be conveniently shifted rightwards to match the master curve at $T_{0}$. The same procedure would be applied to the rest of the short-time relaxation curves in order to get the whole master curve of the relaxation modulus, which characterizes the viscoelastic behavior of the material at $T_{0}$. In this way, the TTS principle can be applied to derive the relaxation modulus of the material for any temperature and for any other time limits than those used in the tests. As a result, the viscoelastic behavior of the material can be determined from a series of short-duration tests performed at distinct temperatures, without requiring an aprioristic definition of the relaxation functions over the time.

In mathematical terms, the TTS principle has been traditionally defined as follows (see Findley et al. [1], Ferry [2] and Lakes [4]):

(1) $$E\left(t;T\right)=E(a_{T}\left(T,T_{0}\right)t;T_{0}),$$

where $a_{T}$ is the so-called shift factor. This equation allows the relaxation modulus *E* of a material at any time *t*, for any temperature *T*, to be obtained from the relaxation modulus at the reference temperature $T_{0}$. In the logarithmic scale, as commonly used, Equation (1) represents a transformation law for the time as given by:

(2) $$\log t_{T}=\log t_{T_{0}}+\log a_{T}\left(T,T_{0}\right).$$

In the assessment of the short-time tests, where the reference temperature is intended to be determined from distinct test temperatures $T_{i}$, Equation (1) can be reformulated as:

(3) $$E\left(t;T_{0}\right)=E\left(a_{T}\left(T_{0};T_{i}\right)t;T_{i}\right),$$

with $T_{i}$ representing the temperature at which the particular *i*-th test is conducted (see Figure 1).

In this paper, the probabilistic basis presumably underlying the relaxation phenomenon will be illustrated and exploited by identifying the relaxation curves at different temperatures with cumulative distribution functions (cdfs) of particular statistical families, such as the normal and the generalized extreme value (GEV) ones. Different origin could be argued for the possible statistical candidate family underlying the viscoelastic phenomenon being studied: the normal distribution could be justified based on the material property derived from the central limit theorem while GEV distributions could be related to the limit behavior of the material for time going to zero and to infinity.

The proposed distributions allow the master curve to be derived by applying the scale transformation property among the cdfs of the mentioned statistical families irrespective of the reference temperature from short-term tests.

The suitability of the proposed approach using normal and GEV cumulative distributions is confirmed when applied to an experimental program consisting in experimental results of relaxation tests on a Polyvinil–Butyral material, commonly referred to as Polyvinil–Butyral (PVB). Fitting of the master relaxation curve and the TTS model is achieved by considering the above-mentioned cdfs directly without the necessity of resorting to classical viscoelastic models, such as the Maxwell generalized by means of Prony series fitting and the WLF-TTS model (see Williams et al. [5]). In this way, the number of parameters chosen is reduced and, consequently, the model, simplified. Furthermore, the identification of the relaxation curves with cdfs implies to take advantage of the experience gained in the transformation of statistical functions both in the analysis and meaning of the model parameters and the transformation law.

## 2. The Current Methodology

As previously mentioned, the TTS principle is widely used in the viscoelastic characterization of materials. However, some of the steps to achieve this goal may influence the results obtained depending on the user’s available experience and criteria applied to the TTS model fitting process (see Knauss [6] and Gergesova et al. [7]). A brief review of the current procedure can be described by next four steps:

1. Derivation of the master curve. The first step consists in deriving the master curve from the experimental data (see Figure 1) by shifting the relaxation curves in the horizontal direction according to the law briefly described in the previous section.
2. Estimation of the experimental shift factors $a_{T}\left(T_{i}\right)$. In the second step, a first approximation of the master curve is achieved, traditionally using manual estimative shifting of the relaxation curves. Thereby, shift factors $a_{T}$ are chosen, which because their arbitrary selection may lead to notably different solutions of the final master curve or even of the TTS model as a whole. Fortunately, in recent years, some new algorithms, such as the CFS algorithm developed by Gergesova et al. [7], allow such a manual shifting to be overcome thus enhancing considerably the accuracy in the derivation of the master curve. The method requires a minimum overlapping among the registered fragments of the relaxation curves to be applied.
  This step 2 implies the analysis of the experimental shift factors $a_{T}$ before fitting the TTS model. One of the main problems is that in general, different trends for the shift factors are noticed depending on them being determined below or above the glass transition temperature $T_{g}$ (see Tanaka [8] and Ferry [2]). Therefore, more than one TTS model is sometimes required to fit adequately the shift factor, as for instance, the Arrhenius model below the $T_{g}$ besides the WLF model (Williams et al. [5]) for the denominated glass transition region (see Ferry [2]). Often, the break-down in the trend of the glass shift factors in the transition region is not clear so that the user has to decide about the limits of the observed trends influencing the accuracy of the TTS fitted models.
3. Fitting the TTS model. The third step implies fitting of the TTS model to be used to predict the viscoelastic behavior at different temperatures. In the case of the WLF model, widely used in the glass transition region (see Williams et al. [5]) and defined as follows:
  (4) $$\log a_{T}\left(T,T_{0}\right)=-\frac{C_{1}^{0}\left(T-T_{0}\right)}{C_{2}^{0}+T-T_{0}},$$
  the constants $C_{1}^{0}$ and $C_{2}^{0}$, being functions of the reference temperature $T_{0}$, must be included in the fitting process with the aim of improving the prediction accuracy (see Ferry [2]). As mentioned previously, most of the times, a second TTS model is usually necessary, i.e., to fit the different shift factor $a_{T}$, either above or below $T_{g}$. This procedure requires a threshold temperature was established to discriminate which one of the two candidate models should be applied. This task is not always straightforward (see Pelayo et al. [9]).
4. Fitting of a viscoelastic model. Finally, the master curve for the relaxation modulus is fitted using a viscoelastic model, such as the generalized Maxwell one. Note that when the classical approach is applied to derive the TTS model, both viscoelastic and TTS model types are implied in the four steps to complete the material characterization.

## 3. The Proposed Methodology

In this section, an alternative approach to the classical one introduced in Section 2 is presented that enables time–temperature conversion among relaxation curves of viscoelastic materials to be accomplished. Some previous considerations concerning dimensional analysis, location and scale transformations and functional equation theory are introduced as necessary concepts for the derivation of the proposed model.

### 3.1. Derivation of the Model

#### 3.1.1. Dimensional Analysis

The use of dimensionless variables, as suggested by the Buckingham theorem (see Buckingham [10]) is recommended as good-practice in the derivation of scientific laws (see Aczél [11], Castillo and Ruiz-Cobo [12] and Castillo et al. [13]). In the present case, in which a derivation of the relaxation function in viscoelastic analysis is envisaged, some previous redefinitions, such as normalization, of the intervening variables are introduced to proceed to the identification with statistical functions.

One the one hand, the relaxation modulus at time *t* is defined as the normalized variable $E^{*}\left(t;T\right)$, by the expression

(5) $$E^{*}\left(t;T\right)=\frac{\log E(t;T)-\log E_{\infty }}{\log E_{0}-\log E_{\infty }}=\frac{\log\left(E\left(t;T\right)/E_{\infty }\right)}{\log\left(E_{0}/E_{\infty }\right)}\in \left[0,1\right],$$

where E(t;T) is the relaxation modulus at the time *t* and temperature *T*, $E_{0}$ is defined as the initial or elastic modulus, while $E_{\infty }$ corresponds with the relaxed viscoelastic modulus for infinite time so that $E_{\infty }\leq E\left(t;T\right)\leq E_{0}$. Unlike to some other authors such as McCrum and Morris [14] and Stouffer and Wineman [15], the initial and relaxed viscoelastic modulus are assumed to be temperature-independent. Note also that the normalization is performed at the logarithmic scale. Because $F\left(t\right)=1-E^{*}\left(t\right)$ is a monotonically increasing function bounded in the interval [0,1], it can be identified, by definition and without loss of generality, as a cumulative distribution function (cdf) and thus handled as such. The same as a cdf, F(t) verifies that $F\left(t_{A}\right)=\mathrm{Pr}\left(t\leq t_{A}\right)$ so that the normalized relaxation modulus $E^{*}\left(t;T\right)$, as defined in (5), represents a survival function of time that satisfy the conditions $\lim_{t\to 0}E\left(t;T\right)=1$ and $\lim_{t\to \infty }E\left(t;T\right)=0$, and can be related to probability. In this sense, the fact that at a certain instant $t_{A}$ the relaxation modulus $E(t_{A};T)$ is equal or greater than a certain value $E_{A}$, such that $E_{\infty }\leq E_{A}\leq E_{0}$, is represented by the normalized relaxation modulus $E^{*}\left(t_{A};T\right)$.

On the other hand, the temperature redefinition requires it to be stable both against location and scale changes (see Castillo and Ruiz-Cobo [12] and Castillo et al. [13]), as happens in the transformation between the conventional temperature systems such as in the Celsius and Fahrenheit. Accordingly, the new dimensionless temperature variable is defined as follows:

(6) $$T^{*}=\frac{T-T_{g}}{T_{r}-T_{g}},\;\;T_{g}<T_{r},$$

where $T_{r}$ can be conveniently defined as the rubbery temperature and $T_{g}$ as the glassy temperature of the material, although any other two reference values could be considered as well. Note that this definition of dimensionless temperature variable avoids the location problem, since it is defined as a quotient of differences.

Finally, since the variable time in viscoelasticity is usually represented in logarithmic scale, its dimensionless redefinition is performed in logarithmic scale as well, which may be easily achieved by considering a suitable constant $t_{0}$, usually measured in seconds, such as $t^{*}=\log\left(t/t_{0}\right)$, with $t_{0}\neq 0$.

#### 3.1.2. Temperature as Scale-Effect

In this model, the effect of temperature on the relaxation modulus is supposed to be represented by a change of the scale parameter of the relaxation curves defined as cumulative distribution functions, according to the stability property of the normal and GEV statistical families. This assumption has been already proposed, in a certain viscoelastic domain, by Gross [16] and successfully applied and confirmed in modeling different parametric effects in materials, such as the length influence in fatigue lifetime prediction of longitudinal elements (see Bogdanoff and Kozin [17], Castillo et al. [18] and Castillo and Fernández-Canteli [19]) and the effect of the test configuration in the failure prediction of laminated glass elements (see Castori and Speranzini [20] and Muñiz-Calvente et al. [21]). Hence, if the relaxation curves are assumed to arise from a minimum principle, then the transformation of the viscoelastic modulus from one temperature to another is defined as follows:

(7) $$E^{*}\left(t^{*};T^{*}\right)=E^{*}{\left(t^{*};T_{0}^{*}\right)}^{Q(T^{*},T_{0}^{*})},$$

which is a functional equation the solution of which is provided by Castillo and Ruíz-Cobo [12] and Castillo et al. [13] (Functional equations are equations in which the unknowns are not variables but functions, and these functions appear as such, i.e., without implying any of their derivatives or integrals):

(8) $$\begin{array}{c} \begin{array}{cc} E^{*}\left(t^{*};T^{*}\right) & =p{\left(t^{*}\right)}^{q(T^{*})},\\ p(t^{*}) & =\exp\left\{-\exp\left[f\left(t^{*}\right)\right]\right\},\\ q(T^{*}) & =\exp\left[-f\left(T^{*}\right)\right],\\ Q(T^{*},T_{0}^{*}) & =q\left(T^{*}\right)/q\left(T_{0}^{*}\right), \end{array} \end{array}$$

where $p(t^{*})$ is a survival function and $q(T^{*})$ is a positive monotonic decreasing function. As previously mentioned, the powerfulness of functional equations is emphasized, since once the model is defined, as for example in Equation (7), all the possible solutions implied are given by solving the functional equation without any additional assumptions related to their functional form.

### 3.2. Proposed Models

#### 3.2.1. The Approach Based on Normal Distribution

**Figure 2:**
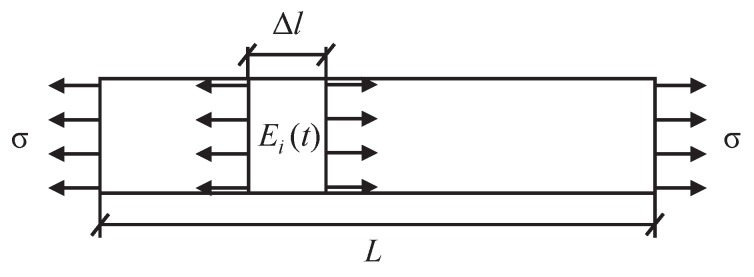
Schematic illustration of a specimen of length *L* subject to a constant stress σ, subdivided in *n* elements of equal length ∆l.

Let us consider a specimen of length *L* supposedly divided into *n* sub-elements of the same length, which are subject to a constant stress σ (see Figure 2). The total length variation occurring in the specimen results as the summation of the local random elongations of each of the *n* sub-elements in which the specimen is virtually subdivided, such that,

(9) $$\delta \left(t\right)=\varepsilon_{T}\left(t\right)L=\frac{\sigma L}{n}\left(\frac{1}{E_{1}\left(t\right)}+\frac{1}{E_{2}\left(t\right)}+\cdots +\frac{1}{E_{n}\left(t\right)}\right)=\frac{\sigma L}{n}\sum_{i=1}^{n}\frac{1}{E_{i}\left(t\right)}=\frac{\sigma L}{E_{T}\left(t\right)}.$$

According to the central limit theorem, it can be stated that the summation of the inverses of the equivalent viscoelastic moduli in Equation (9) for increasing number of elements, i.e., for n→∞ tends asymptotically to a normal distribution irrespective of the random distribution assigned to the equivalent viscoelastic modulus, or reciprocally, the inverse summation of the equivalent viscoelastic moduli must tend to a normal distribution.

Consequently, denoting $H^{*}\left(t^{*};T^{*}\right)={\left[E^{*}\left(t^{*};T^{*}\right)\right]}^{-1}$ as the inverse of the viscoelastic modulus (The inverse of the relaxation modulus, denoted *H* in this work, must not be confused with the corresponding creep compliance *D*), the normalization of the viscoelastic modulus is conveniently achieved for $H^{*}\left(t^{*};T^{*}\right)$ in an equivalent way to Equation (5). Thereafter, assuming that the inverse of the viscoelastic modulus in time follows a normal distribution and the influence of temperature can be assigned to a scale-effect, then the application of the extended normal cumulative distribution function is justified (see Castillo et al. [18] and Castillo and Fernández-Canteli [19]). In this way, the normal model is applied to fit the viscoelastic modulus over time at any temperature:

(10) $${E^{*}\left(t^{*},T^{*};\mu ,\sigma ,\alpha \right)}^{-1}∼{\left[1-N(\mu -\sigma \log Q(T^{*}),\sigma^{2})\right]}^{\alpha },$$

with $-\infty <t^{*}<\infty$ and μ, σ and α being the parameters of the extended normal distribution, respectively. From Equation (10) the horizontal shift over time corresponding with the normal model is calculated as:

(11) $$\log a_{T^{*}}\left(T_{2}^{*},T_{1}^{*}\right)=\sigma \left[\log Q(T_{2}^{*})-\log Q(T_{1}^{*})\right],$$

providing an alternative procedure as that implying the classical shift factor $a_{T}$.

#### 3.2.2. The Approach Based on Extreme Value Distributions

The above considerations justify the theoretical fitting of the E(t) curve using the cumulative distribution function of the normal distribution for this being the most suitable one to describe the evolution of the relaxation modulus as a function of time. Furthermore, the factual existence of the lower bound of time, t=0, points out the convenience of time being measured in a logarithmic scale in order to adapt to the normal distribution. Due to the way data are collected, fitting of both the lower and upper tails of the cdf implies some uncertainty. Results at the lower tail of the fitting function E(t), i.e., for t→0, are not available due to the difficulty of collecting data at very low measurement times, $t<t_{1}$ (see Section 1). In turn, the absence of data at the upper tail of the fitting function E(t), i.e., for $t>t_{2}$ (see Section 1), is assigned to the renouncement to extend the data registration beyond the pre-established upper time limit, $t_{2}$, for the sake of test brevity and program cost reduction. Note that the advantage represented by limiting the tests’ measurement duration of the tests between $t_{1}<t<t_{2}$ implies, as a counterpart, the need of resorting to the time–temperature superposition (TTS) model.

If the characterization of the viscoelastic material points finds subsequently application in a component design implying mainly short-time load acting durations, a suitable estimation of the relaxation modulus at the initial domain becomes relevant. In such a case, the Gumbel distribution for minima, as domain of attraction of the normal or log-normal distribution would be advisable as providing more reliable extrapolating fit at this distribution tail. On the contrary, if the component design is involved with long periods of time, fitting of the upper tail of the cdf, i.e., of the long-time relaxation modulus, can be advantageously achieved by using a Gumbel distribution for maxima (see Galambos [22], Castillo [23] and Castillo et al. [24]).

Assuming that the viscoelastic modulus, E(t), may be approached by a survival minimal Gumbel distribution, then the viscoelastic modulus as a function of time for every temperature is defined by virtue of the stability property, as:

(12) $$E^{*}\left(t^{*},T^{*};\lambda ,\delta \right)=\exp\left[-Q\left(T^{*}\right)\exp\left(\frac{t^{*}-\lambda }{\delta }\right)\right],$$

or equivalently,

(13) $$E^{*}\left(t^{*},T^{*};\lambda ,\delta \right)=\exp\left[-\exp\left[\log Q(T^{*})\right]\exp\left(\frac{t^{*}-\lambda }{\delta }\right)\right],$$

from where it results:

(14) $$E^{*}\left(t^{*},T^{*};\lambda ,\delta \right)=\exp\left[-\exp\left(\frac{t^{*}-\left(\lambda -\delta \log Q(T^{*})\right)}{\delta }\right)\right],$$

with $-\infty <t^{*}<\infty$ and λ and δ as location and scale parameters of the Gumbel distribution, respectively. In fact, a change in the scale parameter due to temperature, such that $\delta (T^{*})$, implies a horizontal translation in time as given by the location parameter, namely:

(15) $$\log a_{T^{*}}\left(T_{2}^{*},T_{1}^{*}\right)=\delta \left[\log Q(T_{2}^{*})-\log Q(T_{1}^{*})\right].$$

This proves that the Gumbel distribution may be an adequate alternative for modeling TTS principle.

**Figure 3:**
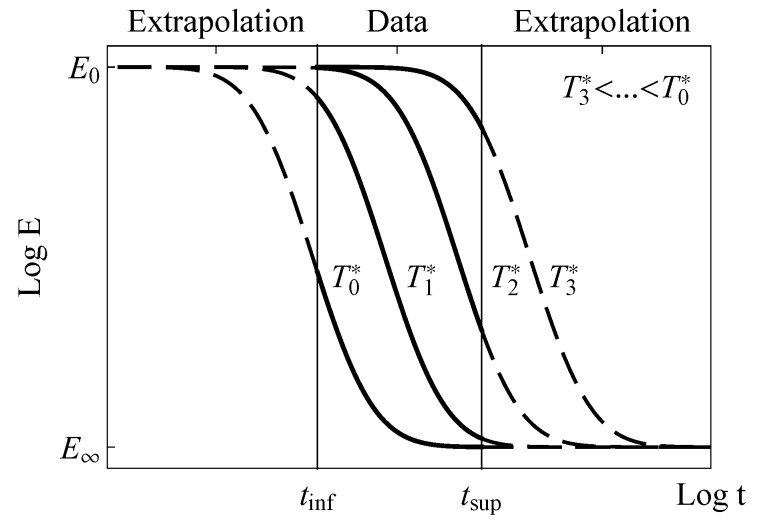
Viscoelastic moduli identified as survival functions for different values of the scale parameter associated with changes of temperature in the Gumbel model, within the experimental window and extrapolated along the time.

The scale-effect for the Gumbel and normal models is shown in Figure 3 both within the experimental window and after extrapolation along the time. In the case of normal distribution, the scale-effect implies a horizontal translation of the cdf, alike the Gumbel distribution, according to Equations (11) and (15), respectively.

### 3.3. Parameter Estimation

Since the values of a lifetime are defined in a deterministic way, the use of the maximum likelihood (ML) estimator is not justified theoretically. For this reason, the probabilistic paper concept lets transform the expression of the normal and Gumbel cdfs in linear forms allowing the parameter estimation to be easily handled with the least squares method (LSM) throughout the experimental data (see Castillo [23] and Castillo et al. [24]). Likewise, the requirements of the validity of LSM technique, as parameter estimation process (see Hadi and Chatterjee [25]), have been verified. Then, the objective minimization functions for the three normal and Gumbel models are derived, respectively as:

(a) Normal model
  (16) $$\min_{\left(\mu ,\sigma ,\alpha ,E_{0},E_{\infty },a_{T_{k}}^{*}\right)}\left\{\sum_{k=1}^{K}\sum_{i=1}^{N}{\left[\frac{\log E_{0}-\log E_{\infty }}{\log E_{ki}-\log E_{\infty }}-{\left[1-N(\mu -\sigma \log Q_{k},\sigma^{2})\right]}^{\alpha }\right]}^{2}\right\},$$
(b) Gumbel model
  (17) $$\min_{\left(\delta ,\lambda ,E_{0},E_{\infty },a_{T_{k}}^{*}\right)}\left\{\sum_{k=1}^{K}\sum_{i=1}^{N}[\frac{\log E_{ki}-\log E_{\infty }}{\log E_{0}-\log E_{\infty }}-{\left[-\exp\left(\frac{\log t_{ik}-\log t_{0}-\left(\lambda -\delta \log Q_{k}\right)}{\delta }\right)\right]]}^{2}\right\},$$

where $E_{k_{i}}$ and $t_{k_{i}}$ are the vectors of the experimental viscoelastic moduli and discrete times, respectively, for each *k*-th temperature, with $Q_{k}=Q\left(T_{k}^{*}\right)$, such that 1≤k≤K. It is worth mentioning that the constants $E_{0}$ and $E_{\infty }$ are also considered as objective variables in the estimation process, providing more robustness to the model. Nevertheless, if only scarce number of experimental data are available in both tails, i.e., at sufficiently high and low temperatures, the estimates of constants $E_{0}$ and $E_{\infty }$ could then been also obtained from the estimation process. Note that the reference value of $t_{0}$ may be fixed arbitrary, for instance as unity, since its consideration is suggested only for dimensional purposes.

As a result of the estimation process, single values for the parameters of each cdf are obtained as opposed to the *k* different values for the *Q* factors. Note that these factors are reduced to only one term (without the corresponding term for reference temperature $T_{0}$) in contrast to their first definition, without loss of generality. In fact, the proposed model does not require a reference temperature so that the shift factor can be used directly to particularize the different expression models to any temperature. This optimization process can be easily codified by an optimization software such as GAMS. As will be shown in the practical example, the *N* points of the *Q* factor resulting from the estimation for each of the tested temperatures must be fitted according to Equation (8) proving experimentally that the shift factors are exponentially distributed, such that:

(18) $$Q\left(T^{*}\right)=\exp\left(\theta_{1}T^{*}+\theta_{0}\right),$$

for any constants $\theta_{1}$ and $\theta_{0}$. Thus, the final expressions for the proposed model are obtained from substituting Equation (18) into the expressions Equations (10) and (14).

### 3.4. Comparison of Errors in Models

In order to compare the absolute errors between the experimental short-term relaxation tests $E_{ki}^{*}$ and their fittings ${\hat{E}}_{ki}^{*}$ for the three models analyzed, the following criterion is suggested:

(19) $$\mathrm{Error}=\sum_{k=1}^{N}\sum_{i=1}^{n}\left|E_{ki}^{*}-{\hat{E}}_{ki}^{*}\right|.$$

## 4. Example of Practical Application

In order to illustrate the usefulness and capability of the proposed approach in practical applications, the methodology is applied to the results from an experimental program on a commercial polyvinil-butyral (PVB), present in manifold applications of different engineering fields such as solar modules and structural laminated glass, among others.

In the following, first, the main characteristics of the experimental program are described. Second, the two proposed models are applied to the assessment of the short-term relaxation test results at different temperatures, and likewise to the determination of the shift factor. Finally, analytical descriptions of E—t field are used to predict the master curves at various temperatures based on the parameter estimation for each model.

### 4.1. Description of the Experimental Program

**Figure 4:**
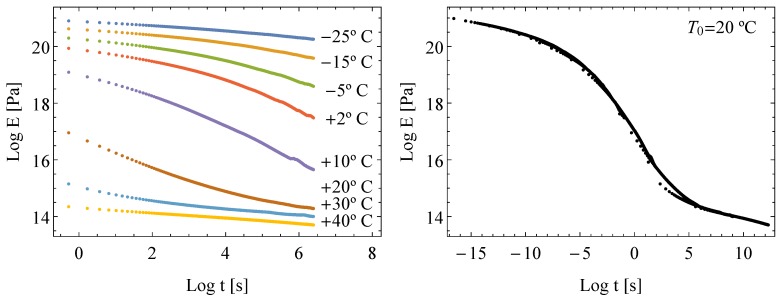
Experimental relaxation curves at different temperatures for the Polyvinil–Butyral (PVB) (**left**), and experimental master curve for 20 °C (**right**).

The experimental program consists in relaxation tests at 8 different temperatures, namely, −25, −15, −5, 2, 10, 20, 30 and 40 °C allowing the temperature-dependent behavior of the material to be reliably captured. Relaxation tests were performed under uniaxial tension with DMA RSA3 equipment of T. A. Instruments provided with a temperature-controlled chamber allowing us to operate under a wide range of temperatures, from −60 °C to 150 °C. The specimens are 25 mm long and 5 mm wide with a thickness of 0.38 mm. The experimental curves for relaxation tests are shown in Figure 4(left). Further details may be found in Pelayo et al. [9].

In order to compare the results obtained with the proposed models, the corresponding experimental master curve for a reference temperature of 20 °C was obtained (see Figure 4 (right)) following the steps described in Section 2.

### 4.2. Short-Term Relaxation Curves Estimation

As described in previous sections, the proposed approach aims at deriving the analytical expression of the whole E—t relaxation field over the whole range of time and temperatures of interest. This happens by estimating the model parameters based on the experimental short-term relaxation curves at different temperatures.

**Figure 5:**
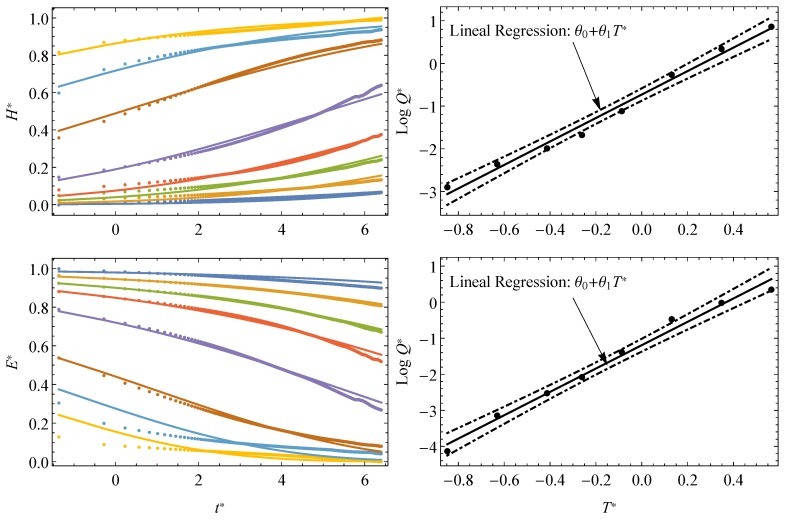
Theoretical predictions and experimental data for relaxation tests on PVB at different temperatures (−25 to 40 °C) using the normal model (**left**) and *Q* factors with 95% confidence intervals (**right**).

In Figure 5 the approach is exemplified by its application to the normal and Gumbel models. As expected, the resulting logarithmic values of the *Q* factor seem to follow a linear trend with respect to the temperature values at the natural scale. An acceptable agreement among the experimental short-term curves and the theoretical curves is confirmed for both models. Certainly, some local discrepancies can be observed in Figure 5, typically at both extreme regions (i.e., E(t=0) and E(t→∞), which, incidentally, are also usually observed in applications of other celebrated conventional T—t models. Disregarding possible uncertainties in the recorded data the assessment of the relaxation modulus in those regions could be improved by proceeding to a local assessment in those regions using the Gumbel model. This could advisable when specific short- or long-term design of the component (i.e., for t→0 and t→∞) is required.

The resulting estimates of the models’ parameters, as provided by the estimation process described in the previous section and the absolute error between the experimental and theoretical results for each model, according to Equation (21), are shown in Table 1. Note the generic notation used in this table where λ and δ represent the location and scale parameters in both normal and Gumbel distributions.

Finally, the expressions of the normal and Gumbel models for the analyzed PVB data, as resulting from the estimates in Table 1, are herewith summarized:

(20) $$E^{*}{\left(t^{*};T^{*}\right)}^{-1}={\left(1-\Phi \left[\frac{t^{*}+16.02T^{*}-18.23}{10.54}\right]\right)}^{11.54},$$

(21) $$E^{*}\left(t^{*};T^{*}\right)=\exp\left[-\exp\left(\frac{t^{*}+18.05T^{*}-3.99}{6.97}\right)\right].$$

### 4.3. Master Curves Prediction

As shown in the preceding section, the experimental short-time relaxation data allows the parameters defining the analytical expressions of E−t field to be determined for the temperatures applied. As an extension, all the master curves can be directly derived from these expressions according to the two proposed models even for a wider interval time as that used in the experimental program. This is because the analytical definition of the shift-factor, according to an exponential law, may be evaluated for any temperature and, subsequently, introduced into the proposed models.

The practical transcendence of this application is emphasized since a unique shift factor definition (given by an exponential law) allows any master curve to be predicted without the limitations due to alternate different laws, such as the Williams–Landel–Ferry and Arrhenius models.

**Figure 6:**
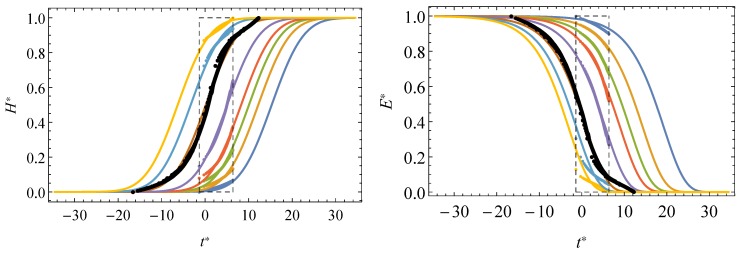
Experimental data for short-term relaxation curves and corresponding master curves provided by the normal model or Gumbel model or using the Williams–Landel–Ferry model.

Figure 6 illustrates the PVB experimental data for the short-time relaxation tests superposed to the resulting master curves.

## 5. Discussion

In summary, this paper attempts at an alternative definition of the TTS principle for building the master curve of a viscoelastic material. Indeed, current methodologies show significant limitations such as the lack of an analytical definition of the E−t field or a required combination of at least two different models for building the master curve. In this way, the proposed model exhibits the following advantages compared with other currently used TTS models:

(a) Dimensional consistency. A robust scientific basis is considered for building TTS models according to good practice in what concerns the dimensional analysis of the involved variables for viscoelastic characterization. As a consequence, the proposed TTS model and their parameters are insensitive to the system of units selected for the fundamental physical magnitudes.
(b) Analytical definition of E−t field. From a practical viewpoint, the analytical definition of the viscoelastic modulus, E(t), as a cdf, represents the most important contribution of the proposed approach allowing the E−t field to be fully defined by fitting the experimental short-term relaxation curves over the whole range of time (i.e., as master curves) and temperatures.
(c) Statistical approach of viscoelastic modulus. The proposed approach contributes to broadening the theoretical framework in which the TTS principle and their models are developed and applied. As a matter of fact, a physical and phenomenological conceptualization of the relaxation phenomenon as statistical cdfs is now available. The already acquired mathematical knowledge about statistical cdfs favors further development of advanced methods for modeling and designing components with viscoelastic behavior.
(d) Independence of the reference temperature. Due to the analytical definition of the E−t field over the whole range of time, the parametric family of master curves are obtained independently of the reference temperature for the first derived of them, satisfying which may be conveniently called a certain uniqueness condition.
(e) Analytical definition of the *Q* shift factor. Classical TTS models are based on discretional assumptions, which allow analytical definitions of the shift factor, *Q*, to be derived, such as the linearity of free-volume concept in the theoretical development of the WLF model. In the new model proposed, the shift factor is derived analytically, free of gratuitous assumptions, based on the theory of the functional equations once the TTS model is defined.
(f) Simultaneous definition of the master curves and short-term curves. The proposed model allows the master curves to be derived directly from the fitting of the experimental short-term relaxation tests, which avoids the hand-made pre-fittings required in the application of current TTS models. Likewise, master curves at different temperatures can be predicted based on the analytical definition of the shift factor, in order to satisfy the uniqueness condition.
(g) Full applicability of the proposed models over all the range of temperatures. The applicability of the displacement factor in current models is limited as the required combination of two models for the definition of this factor depending of the range of temperatures analyzed. On the contrary, the proposed model is applicable over the whole range of temperatures, avoiding different definitions of *Q* for each viscoelastic characterization.
(h) Non-overlapping constraint. The current methodologies require a certain overlapping of the short-time relaxation curves, which must be at least one decade, known as the “rule of thumb” in literature. In turn, the proposed methodology does not require the overlapping among these short-time curves which is an important advantage.
(i) The proposed approach proves to be valid over the whole possible range of temperatures, a question of practical interest, unsatisfactorily accomplished by commonly used TTS methods, such as the Williams–Landel–Ferry model.

Finally, it is worth adding some comments about the suggested use of the extreme value family distributions alternative to the normal one. While the former is justified from a sound physical viewpoint based on strain considerations and application of the limit central theorem, the latter may not respond to that justification.

Nevertheless, the use of the Gumbel distribution in the fitting approach is found to be advisable from a phenomenological point of view when the component design is concerned with the relaxation modulus of the material corresponding to short (maximal value of the relaxation modulus, $E_{0}$), or large (minimal values of the relaxation modulus, $E_{\infty }$) time values, depending on the particular design problem handled. In such cases an asymptotic fitting of both distribution tails using the Gumbel distribution, as representing the domain of attraction of the normal distribution, provides a more reliable prediction of the limiting values $E_{0}$ and $E_{\infty }$ of the relaxation modulus.

Note that the viscoelastic modulus, E(t), for short and large time periods may be the particular, but practical, motivation to apply TTS models in the practical viscoelastic design (see Castori and Speranzini [20] and Muñiz–Calvente et al. [21]), irrespective of the general necessity of characterizing the material over the full range of time.

The three-parameter Weibull distribution was initially assumed in the proposed approach as a feasible alternative for the relaxation modulus, but this option was discarded because of the results in the assessment of the practical example, though acceptable, were less satisfactory than those obtained with the Gumbel distribution. Finally, the results confirm the theoretical equivalence between the Gumbel distribution in the logarithmic scale and the bi-parametric Weibull distribution in natural scale.

## 6. Conclusions

− A previous dimensional analysis of the variables involved in relaxation viscoelastic phenomena provides a robust basis for building TTS models that satisfy the necessary dimensional consistency.
− The proposed methodology allows the analytical expression of the relaxation viscoelastic modulus, E(t), from short to long-term time periods to be derived for any temperature while avoiding the typical hand-made pre-fittings observed by current practical methods.
− Based on a statistical similitude in the evolution of the viscoelastic phenomenon, two suitable models, namely based on the normal and the Gumbel cumulative distribution functions (with possible extension to the bi-parametric Weibull) are proposed, as possible candidates for representing the relaxation function, E(t). In this way the E(t;T) field is analytically defined whereas the temperature effect is found to act as a change in the scale parameter of those distributions.
− The use of the normal model is justified from strain considerations and consequent application of the statistical limit central theorem while the Gumbel model is particularly recommended when maximal or minimal values of the relaxation modulus of the material are determined, as for instance, when design is concerned with short or large loading times. In such cases, asymptotic fitting of the extreme values of the relaxation function based on a Gumbel function provides more reliable parameter estimation.
− Master curves for any temperature other than those used in the test are easily obtained from the analytical definition of the shift factors. In this way, short- and long-term prediction of the relaxation modulus is feasible, irrespective of the temperature history the component is subject to. The functional expression of the latter is derived from functional equations theory without any arbitrary assumption.

## Figures and Tables

**Table 1:** Estimates of dimensionless parameters and the absolute errors for the two proposed models in fitting short-term curves for PVB data.

Model	$\log E_{0}$	$\log E_{\infty }$	λ	δ	α	$\theta_{0}$	$\theta_{1}$	Error
Normal	20.99	13.69	−1.37	10.54	11.54	−1.86	1.52	104.08
Gumbel	21.44	13.69	−1.37	6.97	—	−0.77	2.59	159.95

## Abbreviations

The following abbreviations are used in this manuscript:

E(t;T)	Relaxation modulus as a function of time and temperature
$E^{*}\left(t^{*};T^{*}\right)$	Normalized relaxation modulus as a function of normalized time and temperature
$E_{0}\left(T\right)$	Initial or elastic relaxation modulus
$E_{\infty }\left(T\right)$	Initial or elastic relaxation modulus
*L*	Total specimen length
*t*	Time
*T*	Temperature
$T_{g}$	Transition temperature
$T_{r}$	Rubbery temperature
$T_{0}$	Reference temperature
GEV	Generalized extreme value
TTS	Time–Temperature Superposition
WLF	Williams–Landel–Ferry
TTS	Time–Temperature Superposition
δ(t)	Displacement as a function of time
∆l(t)	Length of each of the *n* subelements in which the specimen of length *L* is virtually subdivided
